# Supplementary material for: The role of dispersal for shaping phylogeographical structure of flightless beetles from the Andes
Source: PeerJ. 2019 Jul 2;7:e7226. doi: 10.7717/peerj.7226 (PMC6611450; doi:10.7717/peerj.7226)
Supplement: Supplemental Information 1 — Outgroups used in this study as also listed in this table. Accession numbers are recorded under each gene column. [file peerj-07-7226-s001.docx]

| **Genus** | **species** | **Code** | **Site** | **Haplotype COI** | **COI** | **Haplotype CAD** | **CAD** | **Reference** |
| --- | --- | --- | --- | --- | --- | --- | --- | --- |
| *Dyscolus* | *alpinus* | SIMT028 | Mojanda | H5 | MK440253 | - | - | This study |
| *Dyscolus* | *alpinus* | SIMT201 | Ruco Pichincha | H1 | MK440254 | H1 | - | This study |
| *Dyscolus* | *alpinus* | SIMT203 | Atillo | H2 | MK440255 | H2 | - | This study |
| *Dyscolus* | *alpinus* | SIMT205 | Atillo | H3 | MK440256 | H4 | MK440262 | This study |
| *Dyscolus* | *alpinus* | SIMT206 | Cayambe | H4 | MK440257 | H3 | MK440263 | This study |
| *Dyscolus* | *alpinus* | SIMT194 | Mojanda | H2 | - | H2 | MK440260 | This study |
| *Dyscolus* | *alpinus* | SIMT196 | Ruco Pichincha | H1 | - | H1 | MK440261 | This study |
| *Dyscolus* | spp. | SIMT208 | Cayambe | H6 | MK440258 | H5 | MK440264 | This study |
| *Dercylus* | *cordicollis* | SIMT260 | Ruco Pichincha | H14 | MK440233 | - | - | This study |
| *Dercylus* | *cordicollis* | SIMT263 | Ruco Pichincha | H15 | MK440234 | H7 | - | This study |
| *Dercylus* | *cordicollis* | SIMT259 | Ruco Pichincha | H14 | - | H7 | MK440245 | This study |
| *Dercylus* | *cordicollis* | SIMT261 | Ruco Pichincha | H14 | - | H6 | MK440246 | This study |
| *Dercylus* | *cordicollis* | SIMT313 | Ruco Pichincha | H14 | - | H5 | MK440247 | This study |
| *Dercylus* | *orbiculatus* | SIMT222 | El Cajas | H5 | MK440224 | H1 | - | This study |
| *Dercylus* | *orbiculatus* | SIMT239 | El Cajas | H6 | MK440225 | H1 | - | This study |
| *Dercylus* | *orbiculatus* | SIMT227 | Culebrillas | H7 | MK440226 | H3 | - | This study |
| *Dercylus* | *orbiculatus* | SIMT229 | Culebrillas | H8 | MK440227 | - | - | This study |
| *Dercylus* | *orbiculatus* | SIMT230 | Culebrillas | H9 | MK440228 | H3 | MK440241 | This study |
| *Dercylus* | *orbiculatus* | SIMT242 | Culebrillas | H10 | MK440230 | H2 | MK440243 | This study |
| *Dercylus* | *orbiculatus* | SIMT244 | Culebrillas | H11 | MK440231 | H1 | - | This study |
| *Dercylus* | *orbiculatus* | SIMT245 | Culebrillas | H12 | MK440232 | H3 | - | This study |
| *Dercylus* | *orbiculatus* | SIMT237 | El Cajas | H5 | - | H1 | MK440242 | This study |
| *Dercylus* | *orbiculatus* | SIMT258 | Culebrillas | H7 | - | H4 | MK440244 | This study |
| *Dercylus* | *praepilatus* | SIMT232 | Salinas | H13 | MK440229 | - | - | This study |
| *Dercylus* | *praepilatus* | SIMT337 | Salinas | H13 | - | H11 | MK440248 | This study |
| *Dercylus* | *praepilatus* | SIMT338 | Salinas | H13 | - | H8 | MK440249 | This study |
| *Dercylus* | *praepilatus* | SIMT234 | Salinas | H13 | - | H13 | MK440251 | This study |
| *Dercylus* | spp. | SIMT212 | Atillo | H1 | MK440220 | H11 | MK440236 | This study |
| *Dercylus* | spp. | SIMT213 | Atillo | H2 | MK440221 | H11 | MK440237 | This study |
| *Dercylus* | spp. | SIMT214 | Atillo | H2 | - | H9 | MK440238 | This study |
| *Dercylus* | spp. | SIMT219 | Atillo | H2 | - | H10 | MK440239 | This study |
| *Dercylus* | spp. | SIMT216 | Atillo | H3 | MK440222 | H12 | MK440250 | This study |
| *Dercylus* | spp. | SIMT221 | Atillo | H4 | MK440223 | H9 | - | This study |
| *Dercylus* | spp. | SIMT265 | Cotacachi | H16 | MK440235 | H5 | MK440252 | This study |
| Dercylus | spp. | SIMT220 | Atillo | H2 | - | H8 | MK440240 | This study |
| *Agonum* | *aeruginosum* | SIMT323 | Cherry Farm, SC, USA | Outgroup | MK457691 | Outgroup | MK457695 | This study |
| *Agonum* | *extensicolle* | SIMT322 | Ellicot rock, SC, USA | Outgroup | MK457692 | Outgroup | MK457696 | This study |
| *Amara* | *apicaria* | SIMT334 | Clemson, SC,USA | Outgroup | MK457690 | Outgroup | MK457694 | This study |
| *Dicaelus* | *elongatus* | SIMT329 | Sand Hills, SC, USA | Outgroup | MK879651 | Outgroup | - | This study |
| *Incagonum* | *aeneum* | SIMT326 | Releche, Chimborazo | Outgroup | MK457693 | Outgroup | MK457697 | This study |
|  |  |  |  |  |  |  |  |  |
| *Loxodactylus* | *carinulatus* | KF551775 | Australia | Outgroup | KF551775 | Outgroup | KF551654 | Will, K.W. 2015. New species of *Notonomus* Chaudoir, 1862 (Coleoptera, Carabidae) and  a redefinition of the genus based on a molecular phylogenetic  analysis (unpublished) |
| *Notonomus* | *sp.* | KF551785 | Australia | Outgroup | KF551785 | Outgroup | KF551664 | Will, K.W. 2015. New species of N*otonomus* Chaudoir, 1862 (Coleoptera, Carabidae) and  a redefinition of the genus based on a molecular phylogenetic  analysis (unpublished) |
| *Oodes* | *amaroides* | SIMT328 | Cherry Farm, SC, USA | Outgroup | MK879650 | Outgroup | - | This study |
| *Platynus* | *decentis* | KR604910 | McLean Bog Natural, NY, USA | Outgroup | - | Outgroup | KR604910 | Will,K.W. 2016. Molecular phylogenetics of the ground beetle tribe Platynini (unpublised manuscript). |
| *Platynus* | spp. | SIMT332 | Sand Hills, SC, USA | Outgroup | MK440259 | - | - | This study |
| *Sarticus* | *obesulus* | KF551801 | Australia | Outgroup | KF551801 | Outgroup | KF551677 | Will, K.W. 2015. New species of *Notonomus* Chaudoir, 1862 (Coleoptera, Carabidae) and  a redefinition of the genus based on a molecular phylogenetic  analysis (unpublished) |
